# Extracting Structural Information from Physicochemical Property Measurements Using Machine Learning—A New Approach for Structure Elucidation in Non-targeted Analysis

**DOI:** 10.1021/acs.est.3c03003

**Published:** 2023-09-25

**Authors:** Dimitri Abrahamsson, Christopher L. Brueck, Carsten Prasse, Dimitra A. Lambropoulou, Lelouda-Athanasia Koronaiou, Miaomiao Wang, June-Soo Park, Tracey J. Woodruff

**Affiliations:** 1 Department of Pediatrics, New York University Grossman School of Medicine, New York, New York 10016, United States; 2 Department of Obstetrics, Gynecology and Reproductive Sciences, Program on Reproductive Health and the Environment, University of California, San Francisco, California 94107, United States; 3 Department of Environmental Health and Engineering, Johns Hopkins University, Baltimore, Maryland 21205, United States; 4 Exponent, Environmental and Earth Sciences Practice, Bellevue, Washington 98007, United States; 5 Risk Sciences and Public Policy Institute, Bloomberg School of Public Health, Johns Hopkins University, Baltimore, Maryland 21205, United States; 6 Department of Chemistry, Aristotle University of Thessaloniki, University Campus, 54124 Thessaloniki Greece; 7 Department of Toxic Substances Control, Environmental Chemistry Laboratory, California Environmental Agency, Berkeley, California 94710, United States; 8 Laboratory of Environmental Pollution Control, Department of Chemistry, Aristotle University of Thessaloniki, GR-541 24 Thessaloniki, Greece; 9 Center for Interdisciplinary Research and Innovation (CIRI-AUTH), Balkan Center, Thessaloniki, GR-57001, Greece

**Keywords:** non-targeted analysis, machine learning, physicochemical properties, structure elucidation

## Abstract

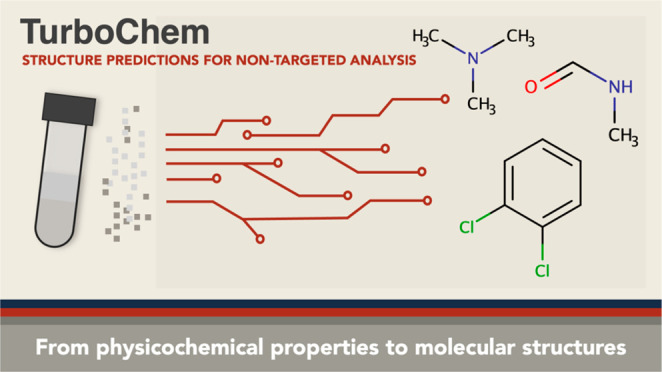

Non-targeted analysis (NTA) has made critical contributions in the fields of environmental chemistry and environmental health. One critical bottleneck is the lack of available analytical standards for most chemicals in the environment. Our study aims to explore a novel approach that integrates measurements of equilibrium partition ratios between organic solvents and water (*K*_SW_) to predictions of molecular structures. These properties can be used as a fingerprint, which with the help of a machine learning algorithm can be converted into a series of functional groups (RDKit fragments), which can be used to search chemical databases. We conducted partitioning experiments using a chemical mixture containing 185 chemicals in 10 different organic solvents and water. Both a liquid chromatography quadrupole time-of-flight mass spectrometer (LC-QTOF MS) and a LC-Orbitrap MS were used to assess the feasibility of the experimental method and the accuracy of the algorithm at predicting the correct functional groups. The two methods showed differences in log *K*_SW_ with the QTOF method showing a mean absolute error (MAE) of 0.22 and the Orbitrap method 0.33. The differences also culminated into errors in the predictions of RDKit fragments with the MAE for the QTOF method being 0.23 and for the Orbitrap method being 0.31. Our approach presents a new angle in structure elucidation for NTA and showed promise in assisting with compound identification.

## Abstract 2

The lack of commercially available analytical standards for most chemicals in the environment presents a critical bottleneck in detecting and measuring organic contaminants in the environment and in humans. This study presents a novel approach for extracting structural information from physicochemical property measurements using machine learning for chemicals detected through non-targeted analysis.

## 1 Introduction

Non-targeted analysis (NTA) and untargeted metabolomics have made critical contributions to our understanding of environmental chemical exposures and the development of human disease.^1−4^ Despite these advancements, our understanding of the role of endogenous and exogenous small molecules in the development of human disease remains limited, especially in comparison to the great advances made in characterizing the human genome and proteome.^5^ This gap in understanding is in part due to limited analytical and computational methods for studying the exposome and the metabolome. However, recent technological advances in high-resolution mass spectrometry (HRMS) with benchtop instruments such as Orbitrap and quadrupole time-of-flight (QTOF) mass spectrometers have sparked broad interest for their potential to discover previously unknown chemicals and shed light on the intricate relationship between environmental chemical exposures and biological outcomes.

The application of HRMS instruments in the agnostic study of environmental chemical exposures, commonly termed NTA, enables us to capture new and lesser-known molecules that would have previously remained undetected with conventional targeted analytical techniques. However, the scope of mainstream NTA is often limited, as unambiguous chemical identification ultimately depends on the availability of analytical standards for annotation confirmation. Nuñez et al.^5^ estimated that out of about 1,000,000 chemical compounds that are listed as chemicals of environmental importance on EPA’s CompTox Chemicals Dashboard (from here on referred to as the “Dashboard”), less than 2% are available as analytical standards. This practically means that all chemical measurements to date, including all environmental, human exposure, and epidemiological studies, are concentrated in chemical space leaving out 98% of environmental chemicals that could potentially have a negative impact on the environment and human health.

One of the main reasons behind this discrepancy is that chemical manufacturers in the U.S. are generally not required to provide analytical standards for the chemicals that they manufacture and release to the environment.^6^ The only category of chemicals for which they are required to submit analytical standards is pesticides, of which residues are commonly found in foods.^6^ This constitutes a critical obstacle in the study of the environmental chemistry of organic contaminants and the study of the exposome and prevents researchers from identifying and quantifying chemicals that lack analytical standards in environmental and biological samples. A change in the *status quo* would require major policy changes, which, although necessary, are not expected to occur any time soon.

As a result, while important advances have been made in the field, the number of identified compounds in NTA studies often does not exceed 5% of the number of detected chemical features (masses and retention times) in environmental and biological samples.^1,7−9^ There is thus a need to develop new computational approaches that can provide structural information about detected chemical features without completely relying on analytical standards. Importantly, while identification with analytical standards remains the gold standard, it cannot serve as a viable path for comprehensive characterization of the chemical space. Computational approaches have shown great promise in structure elucidation through *in silico* structure predictions for NTA.^10,11^ With such approaches, one could compose a diagnostic image for a detected molecule by using information from multiple independent sources as layers of evidence in place of analytical standards.

**Figure 1:**
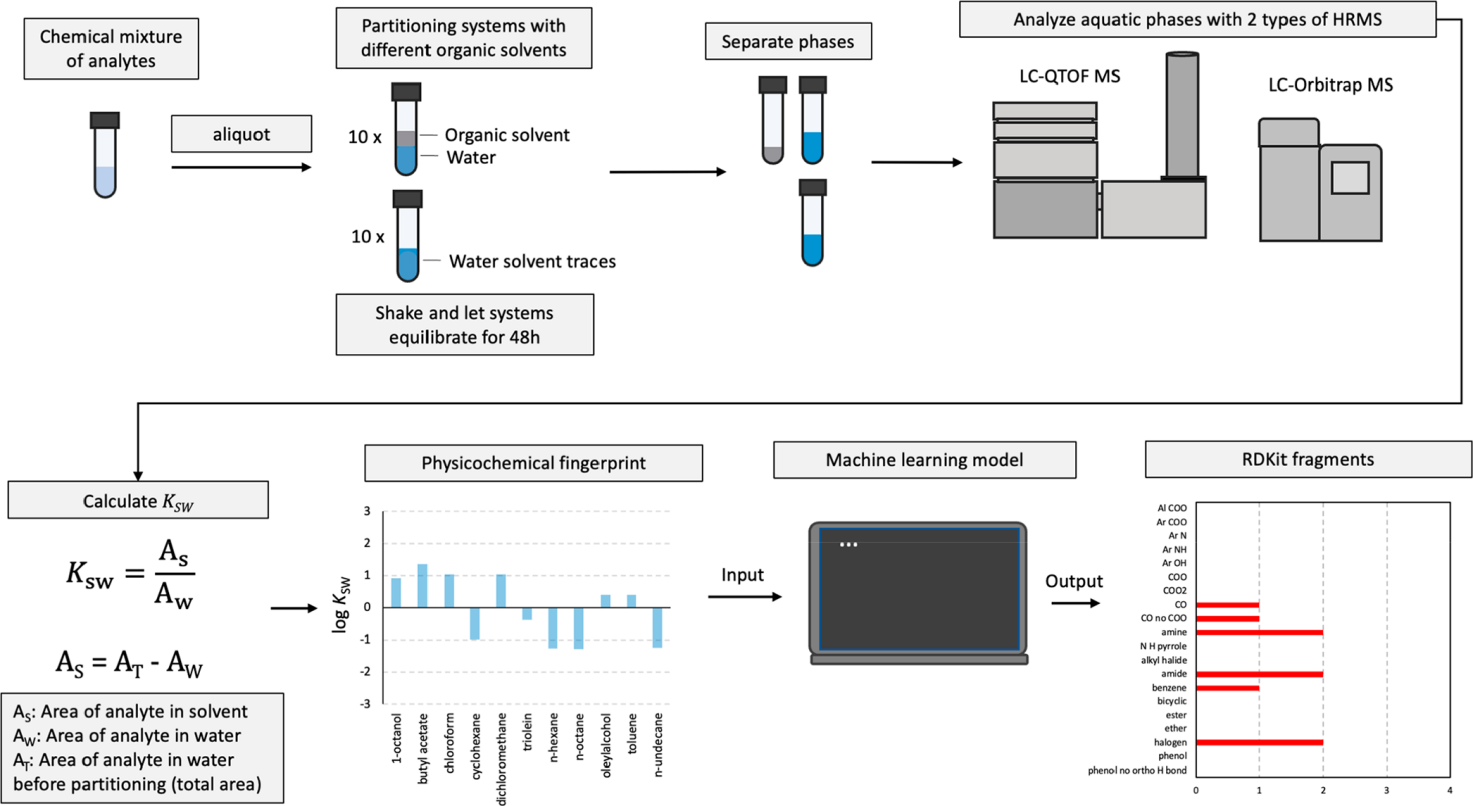
Experimental and computational workflow for obtaining *K*_SW_ measurements for detected chemical features through high-resolution mass spectrometry and converting them into RDKit fragments with a machine learning model. The fragments can then be used to search chemical databases for compounds that match the description, as one would when searching with MS/MS spectra.

In our previous study,^11^ we explored *in silico* the potential of integrating physicochemical property measurements to predictions of molecular structures for chemical features detected by NTA. These physicochemical properties were equilibrium partition ratios between organic solvents and water (*K*_SW_). As these properties, are often sufficiently different among different isomers, each isomer has a unique combination of these properties, which we refer to as the “physicochemical fingerprint” (Figure 1). We evaluated the potential of the physicochemical fingerprint to be used as a signal, which with the help of a machine learning algorithm can be translated into a series of molecular fragments or functional groups (e.g., OH, benzene rings, ether groups, COOH etc.), which then in turn can be used to search databases for molecules that match to that series. Our previous study evaluated the computational aspects of that method (model training and predictions) and showed an average expected accuracy of about 70% at predicting the right molecular structure.

In this study, we focused on evaluating both the computational and experimental aspects of the workflow. When considering the experimental aspects of the workflow, we need to note that different types of mass spectrometry instrumentation may influence the accuracy of the method. Two of the most common types of benchtop mass spectrometers are quadrupole time-of-flight mass spectrometry (QTOF MS) and Orbitrap MS. Two critical distinctions between the two instruments are their mass resolution and their dynamic range (defined as the ratio between the minimum and the maximum concentration that can be detected simultaneously in a sample). Based on data from the manufacturers of the two instruments, Thermo Scientific^12^ and Agilent,^13^ an Orbitrap is expected to have a mass resolution of 200,000 at an *m*/*z* of 300, while a QTOF is expected to have a mass resolution of 40,000 for the same *m*/*z*. Differences in mass resolution could potentially affect our calculations if the instrument is not able to distinguish between chemicals with very similar masses. Overlap in detected monoisotopic masses would result in overlapping peaks, which would in turn result in erroneous calculations of *K*_SW_.

When discussing dynamic range, it is important to mention that there are two types of dynamic range: the intrascan dynamic range and the interscan dynamic range.^14^ The intrascan dynamic range is defined as the abundance ratio between the minimum and maximum detected abundance within a specific spectrum.^14^ The interscan dynamic range is defined as the abundance ratio between the minimum and maximum detected abundance of all recorded scans across a chromatogram.^14^ In a previous study, Kaufmann and Walker^14^ observed that Obritraps showed a narrower intrascan dynamic range compared to QTOFs, likely due to the limited physical capacity of the C-trap and Orbitrap components of the instrument. Differences in the interscan dynamic range would likely affect the peak areas of the detected chemical features, which would in turn affect the calculations of *K*_SW_.

The aim of this study is to evaluate the feasibility of the experimental and computational aspects of the method (measurements of *K*_SW_) using a chemical mixture containing 185 chemicals and two different instruments an LC-QTOF MS and an LC-Orbitrap MS, and to assess the accuracy of the previously developed algorithm^11^ at predicting the correct chemical structure. Employing two different instruments helped us evaluate the reproducibility of the approach across different platforms and assess whether the findings are influenced by the instrument type.

## 2 Materials and Methods

### 2.1 Workflow

**Figure 2:**
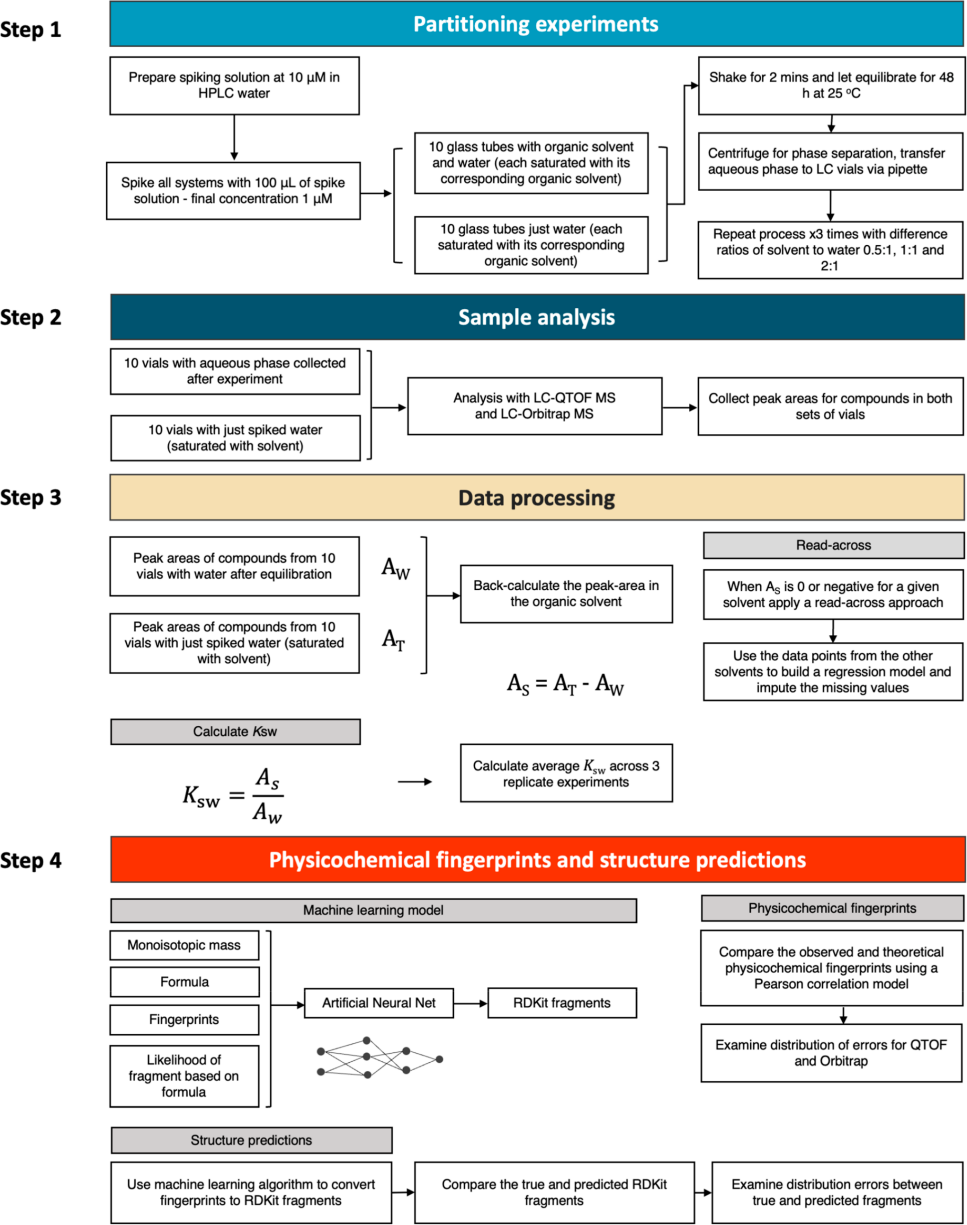
Flowchart diagram describing the experimental and data processing steps involved in this study.

The key components of the experimental and computational aspects of our workflow are presented in Figure 1 and the individual steps for each component are present in the flowchart in Figure 2.

### 2.2 Experimental Section

#### 2.2.1 Equilibrium Partitioning

The equilibrium partitioning ratios were conducted using the shake flask method following the OECD guidelines^15^ for measuring partition ratios between organic solvents and water. The organic solvents used in the partitioning experiments were 1-octanol, butyl acetate, chloroform, cyclohexane, dichloromethane, *n*-hexane, *n*-octane, oleyl alcohol, toluene, and n-undecane (Sigma-Aldrich). All solvents were preconditioned with water, and all water samples were preconditioned with their corresponding solvents prior to the experiments. This is a standard step in octanol–water experiments, and it is meant to address any measurement uncertainties that can occur when small amounts of minimally water-soluble solvents like octanol dissolve in water and when small amounts of water dissolve in the organic phase.^15^ This step is of smaller importance in organic solvents such as hexane that are not at all miscible in water. Briefly, 10 mL of solvent and 10 mL of HPLC water were added to a small round flask (10 flasks in total), and the flasks were gently mixed and were allowed to equilibrate for 24 h.

The analytes used in the partitioning experiments were offered by the U.S. EPA for the purposes of this study and were developed during the EPA’s Non-Targeted Analysis Collaborative Trial (ENTACT). The preparation of the chemical mixtures is described in detail in the study of Ulrich et al.^16^ For the purposes of this study, we used mixture 504 which contained 185 chemical compounds. The chemical structures and the chemical identifiers of the compounds in the mixture are presented in Supplemental Spreadsheet 1. The mixture was diluted from 20 mM first with methanol and then with HPLC water in a series of dilutions to a concentration of 10 μM and was used as a spiking solution. Each partitioning system was prepared by transferring 0.9 mL of water (preconditioned with its corresponding organic solvent) and 1 mL of organic solvent (preconditioned with water) to a test tube and adding 100 μL of the spiking solution to the aquatic phase in order to reach a starting concentration of 1 μM in water. The test tubes were shaken using a vortex shaker for 2 min and then left to equilibrate for 48 h. Alongside the 10 partitioning systems, another set of 10 water samples were prepared by transferring 0.9 mL of water preconditioned with its corresponding organic solvent to a test tube and spiking it with 100 μL of the spiking solution, herein known as water controls. The purpose of the water controls was 2-fold: (i) to determine the initial peak area of the analyte in water prior to equilibration and (ii) to account for differences in ionization efficiency due to the presence of traces of organic solvents in the water. The experiments were done in triplicates with varying ratios of organic solvent to water as recommended by the OECD guidelines,^15^ 1:1, 2:1, and 0.5:1, respectively, for experiments 1, 2, and 3.

After equilibration, the 10 test tubes with the organic solvents and water were centrifuged at 3000 rpm for 10 min at room temperature to improve the separation of the two phases. An aliquot of 500 μL was taken from the aquatic phase with a Pasteur pipet and was transferred to an LC vial. Similarly, all water controls were transferred to LC vials and stored at −20 °C prior to analysis. Each replicate experiment was accompanied by one water blank (HPLC water) that followed the same procedure as that of the samples. The total number of samples was as follows: 10 samples from the partitioning experiments × 3 replicates = 30, and 10 water controls × 3 replicates = 30, plus 1 water blank × 3 replicates = 3; total number of samples = 63.

#### 2.2.2 Instrumental Analysis

The instrumental analysis of the samples was conducted in two different types of instruments an Agilent 1290 ultrahigh-performance liquid chromatography (UPLC) coupled to an Agilent 6550 quadrupole time-of-flight (QTOF) mass spectrometer and a Thermo Scientific

RSLCnano UPLC system coupled to a Q Exactive HF high-resolution mass spectrometry Orbitrap mass spectrometer.

##### 2.2.2.1 Analysis Using LC-QTOF MS

##### Chromatography

An Agilent 1290 UPLC with an Agilent Eclipse Plus C18 column (2.1 × 100 mm, 1.8 μm) was used for the chromatographic separation of the analytes. The mobile phase consisted of two solutions: (A) 5 mM ammonium acetate (Sigma-Aldrich, ≥98%) in HPLC water (Sigma-Aldrich, ≥99.5%) with 0.1% MeOH and (B) 5 mM ammonium acetate in methanol (MeOH; Sigma-Aldrich, ≥99.9%) with 10% HPLC water, which were mixed under the following gradient program: 0 min 10% B and 90% A, 0–15 min gradual increase to 100% B, 16–20 min equilibration at 100% B. All samples were analyzed in duplicate injections, and water blanks were analyzed in the beginning of each batch.

##### Mass Spectrometry

The Agilent 6550 QTOF was operated in both positive and negative electrospray ionization modes (ESI+ and ESI-) to acquire full scan mass spectra (MS) in the range of 100–1000 Da with a resolving power of 40,000 and a mass accuracy <5 ppm. The instrument was calibrated before analyzing each batch and the mass accuracy was corrected with reference standards using references masses 112.985587 and 1033.988109 for negative ionization mode and 121.050873 and 922.009798 for positive ionization mode.

##### 2.2.2.2 Analysis Using LC-Orbitrap MS

##### Chromatography

A Thermo Scientific RSLCnano ultrahigh-performance liquid chromatography system was used for analyte separation. The mobile phase was 5 mM ammonium acetate (LC-MS grade, Fisher) in Milli-Q water with 0.1% MeOH (LC-MS grade, Fisher) (A) and 5 mM ammonium acetate in MeOH with 10% Milli-Q water (B) mixed according to the following gradient program: 0–1 min, 10% B; 1–16 min, 10–100% B; 16–21 min, 100% B; 21–30 min, 10% B. The column used was a Zorbax RR Extend-C18 (Agilent) with 150 mm length, 1 mm diameter, 3.5 μm particle size, and 80 Å pore size. The column temperature was maintained at 35 ± 5 °C. Sequence injection order for the samples was randomized, and all samples were injected in duplicate.

##### Mass Spectrometry

A Thermo Scientific Q Exactive HF high-resolution mass spectrometry Orbitrap system operated with electrospray ionization in polarity switching mode with a resolution of 60,000, an automatic gain control target of 3e6, a maximum injection time of 200 ms, and a scan range of 100–1,000 *m*/*z*. The instrument was calibrated weekly to ensure a good performance.

### 2.3 Data Collection and File Processing

#### 2.3.1 LC-QTOF MS Data Files

The collected datafiles with the total ion chromatograms were processed with MS-DIAL^17^ which is an open-source software that was developed by UC Davis and RIKEN (Japan). The features were aligned across samples, and they were matched to the monoisotopic masses of the chemicals contained in the mixture (Supplementary Spreadsheet 1) within a 10 ppm mass difference. Those chemical features whose peak areas were at least 1.3 times higher in the samples compared to the blanks were considered to be true positives. All MS-Dial processing parameters are presented in Supplementary Spreadsheet 3.

#### 2.3.2 LC-Orbitrap MS Data Files

Data generated using the Orbitrap method were also processed with MS-Dial. The detected features were aligned across samples and matched to the monoisotopic masses of the chemicals in the mixture (Supplementary Spreadsheet 1) within a 5 ppm mass difference. Those chemical features whose peak areas were at least 1.3 times higher in the concentrated stock solution compared to the peak areas in the samples were considered true positives. All MS-Dial processing parameters are presented in Supplementary Spreadsheet 3.

### 2.4 Calculation of *K*_SW_

Conventionally, *K*_SW_ is calculated as
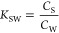
1where *C*_S_ is the concentration of the analyte in the organic solvent and *C*_W_ is the concentration of the analyte in the aquatic phase.^18^ Considering that the concentrations of chemicals in NTA are unknown, we can rewrite the equation in terms of peak areas:
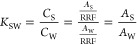
2where *A*_S_ is the peak area of the analyte in the organic solvent, *A*_W_ is the peak area of the analyte in the water, and RRF is the relative response factor of the analyte. It is important to note that this equation expressed in peak areas assumes that the analytes in the sample are within the linear range of the calibration curve.

In order to minimize matrix effects associated with the organic solvents, in our study, we analyzed only the aquatic phases, and we calculated *A*_S_ as follows:
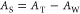
3where *A*_T_ is the total area of the analyte measured in the water controls. As described above, water control samples were prepared using HPLC water saturated with its corresponding solvent and spiked with the chemical mixture.

### 2.5 Read-Across Imputation

In cases where *A*_S_ ≤ 0, we applied a read-across imputation approach by inferring *K*_SW_ for a particular solvent from the *K*_SW_ values of the other solvents for the same analyte. This imputation step is necessary in order for the data set to be used as input in the machine learning model (described below) and in order for the model to make structural predictions. Since the model is trained on 10 solvent systems, all 10 values are required for each chemical for the model to be able to make predictions. Missing values in one or more of the solvent systems will result in Not a Number (NaN) values for the RDKit fragments. This imputation approach is based on the assumption that the *K*_SW_ for one solvent (e.g., hexane) can be described as a function of *K*_SW_ values from 3 or more other solvents (e.g., toluene, octanol, and octane) for the same analyte by a multilinear regression model:
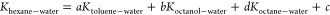
4where *c* is a constant and *a*, *b*, and *d* are the weights of *K*_toluene–water_, *K*_octanol–water_, and *K*_octane–water_, respectively.

Eq 4 can be written in its generalized form as
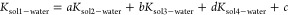
5

To apply the described approach in our study, we started by utilizing the curated version of the Blood Exposome database^19^ that we published in our previous study^11^ which contained 18,973 compounds that have been previously reported in human blood and their *K*_SW_ values which we downloaded from the UFZ-LSER database.^20^ The data collection and curation process is described in detail in our previous study.^11^ In this study, we enriched the database with additional chemicals from the Dashboard to a final number of 32,191 chemical structures. To do this, we searched the Dashboard for all chemical structures that corresponded to the molecular formulas in the mixture and all chemicals whose monoisotopic masses were within 10 ppm of the chemicals in the mixture. The purpose of this step is to enrich the database and, by extension, the training set of the model so that it can make more accurate predictions. The updated version of the database, referred to as “TurboChemDB” here on, was used to build multilinear regressions where each *K*_SW_ is described as a function of three other *K*_SW_. The database is provided in Supplementary Spreadsheet 2. The process was automated using a Python script, which (i) screened the experimental data set for missing *K*_SW_, (ii) went back to TurboChemDB and constructed multilinear regressions for that *K*_SW_ by randomly selecting three other *K*_SW_ as described in eq 4 and determining the coefficients (a, b, and d) and the constant (c) using least-squares minimization. The responses (*K*_sol1–water_) were predicted 100 times after randomly sampling different solvent–water equilibrium partitioning ratios (predictors; *K*_sol2–water_, *K*_sol3–water_, and *K*_sol4–water_). The predictions were then averaged across the 100 iterations and were used to impute the missing values in the data set. The script for the regression model together with all the code developed in this study are available on GitHub (https://github.com/dimitriabrahamsson/turbo-chem).

### 2.6 Evaluating the Physicochemical Fingerprint Measurements

We focused our evaluation on a subset of chemicals whose physicochemical fingerprints showed good agreement (Pearson *R*^2^ ≥ 0.8) between the QTOF and Orbitrap methods, regardless of their agreement with the theoretical values from the UFZ-LSER database. This allows us to simulate how our approach would be applied in the real world to a set of environmental or biological samples when we would not know the chemical structures of the detected chemicals. We then used a Pearson regression model to compare the observed physicochemical fingerprints from both experimental methods to the theoretical fingerprints from the database. We calculated the coefficient of determination (*R*^2^) between the experimental and theoretical *K*_SW_ values and evaluated the distribution of the *R*^2^ values. In this approach, the experimentally determined physicochemical fingerprint of each chemical is represented as a set of values (*n* = 11) stored as an array (e.g., [2.4, 6.3, ... 5.9]) and it is used as the *x* variable in the regression model. Similarly, the theoretical fingerprint of each chemical is represented in the same way, and it is used as the *y* variable.

At this stage, it is important to note that for ionic chemicals in our samples what we actually measure in the partitioning experiments is the distribution coefficient or distribution ratio (*D*_SW_). Considering that the TurboChem database contains only values for *K*_SW_ and the machine learning model described in the sections below is trained on *K*_SW_ values, applying the model directly to *D*_SW_ would not be appropriate. In the section below, we explain how we can utilize *D*_SW_ measurements and transform them in a way that they can be interpreted by the model. *D*_SW_ is defined as
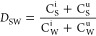
6where, C^i^ is the concentration of the ionized species of the analyte and C^u^ is the concentration of the un-ionized species of the analyte in the organic solvent (subscript S) and in the aquatic phase (subscript W). Considering that the concentration of ionic species in organic solvents is negligible compared to the concentration in the aquatic phase, eq 6 can be simplified as
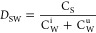
7*D*_SW_ can be described in terms of peak areas as follows:
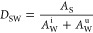
8

As the concentration of ionized and un-ionized species depends on the pH of the solution and on the dissociation constant (p*K*_a_) of each molecule, *D*_SW_ is different at different pH values. For example, the *D*_OW_ of molecule M will be different at pH = 2 and at pH = 8. However, as the concentration of the ionized species (M^+^ or M^–^) is controlled only by the aquatic phase, the difference between *D*_octanol–water_ and *D*_hexane–water_ should remain the same regardless of pH. So, while the absolute values of *D*_octanol–water_ and *D*_hexane-water_ are going to be different at pH = 2 and pH = 8, the differences between *D*_octanol–water_ and *D*_hexane-water_ are going to be the same. This can be described as
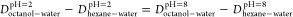
9

By extension, following the same principle, we can assume that these differences are also the same in *K*_octanol–water_ and *K*_hexane–water_:
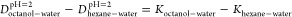
10

This is controlled in our algorithm by employing a standard scaler that standardizes the *K*_SW_ across all solvents by removing the mean and scaling to unit variance. The scaler is applied to both the experimental *D*_SW_ and theoretical *K*_SW_ values in the database. This step ensures that, for the model training and predictions, we are using the differences between the various *D*_SW_ and *K*_SW_, and not the absolute measurements. Thus, when we refer to poststandardization *D*_SW_ values, we use the notation *K*_SW_.

### 2.7 Predictions of Molecular Fragments and Evaluation

#### 2.7.1 Model Design and Implementation

A machine learning model developed and evaluated in our previous study^11^ was used to convert the physicochemical fingerprints into molecular fragments. The model is built as an artificial neural network (ANN) using TensorFlow^21^ as the machine learning platform and Python^22^ as the programming language. The model uses as inputs the physicochemical fingerprints represented as arrays (e.g., [1.2, 0.4, −3.0, ... 3.1]) along with the monoisotopic mass and the molecular formula for each chemical, and outputs the presence and number of RDKit fragments,^23^ which are functional groups and substructures, such as benzene rings, ether groups, alcoholic groups, etc. The network was composed of 1 input layer, 10 hidden layers with 500 nodes in each layer with a rectified linear unit (ReLu) as the activation function, 1 dropout layer to control for overfitting, 1 final hidden layer with 500 nodes using an exponential activation function, and 1 output layer. The optimizer was Adamax and the optimization step was set to 0.001. The model was trained using TurboChemDB for 200 epochs, and it was evaluated using an 80/20 split and a shuffle-split 5-fold cross validation. The weights and biases of the ANN were optimized by minimizing the mean absolute error (MAE) for the predictions in the training set and testing them on the testing set.

In this study, we modified the input parameters of the model to also include a list of the most likely RDKit fragments for each molecular formula. This was done by (i) searching the Dashboard and collecting all available isomers for each molecular formula that was present in TurboChemDB, along with the metadata for each isomer using a parameter called Data Sources,^24^ and their canonical SMILES; (ii) we then collected all their RDKit fragments and ranked all isomers for each formula by their Data Sources; (iii) we normalized the number of Data Sources for each formula from 0 to 1 so that each isomer had a corresponding number from 0 to 1 depending on the number of Data Sources; (iv) finally, we multiplied the number of RDKit fragments for each isomer with the normalized number for Data Sources and calculated the sum for each RDKit fragment per molecular formula. This final number represents the likelihood of each RDKit fragment being present in a molecular structure with a given formula. This addition was done to focus the predictions of the model on a reasonable range of values based on known molecular structures.

The model was then used to make predictions for the set of chemicals that was selected in the previous step. The predicted RDKit fragments were compared against the true RDKit fragments using a Pearson regression model and calculating the *R*^2^ and MAE. To clarify, true RDKit fragments are the true RDKit fragments (substructures) contained in molecule, for example for 1,4-dichlorobenzene, 1 benzene ring, and 2 halogens; whereas, predicted RDKit fragments are the substructures that are predicted from the machine learning model for a detected chemical feature given a specific monoisotopic mass and a physicochemical fingerprint. Finally, we evaluated the distribution of *R*^2^ and MAE and examined which chemicals were predicted with high accuracy and which with poor accuracy.

## 3 Results and Discussion

### 3.1 Compound Detection

Out of 185 compounds in the mixture, 44 compounds were detected with the QTOF method, and 113 were detected with the Orbitrap method (Supplementary Spreadsheet 3). After calculating the log *K*_SW_ for all the compounds and all solvent systems, 16 compounds showed an agreement of *R*^2^ ≥ 0.8 between the log *K*_SW_ measurements in the two data sets (Table S1). We should note at this point that the number of detected compounds is rather low compared with the number of compounds in the mixture, especially for the QTOF method. However, it is worth noting that these chemical mixtures were designed to be challenging as part of the ENTACT trial to test the limits of analytical methods. In addition, many of the compounds in the mixture may not be LC-amenable as these mixtures were not designed with a specific method in mind but rather to be challenging and to cover as much of the chemical space and analytical methods possible.^16,25^

### 3.2 Physicochemical Fingerprints

**Figure 3:**
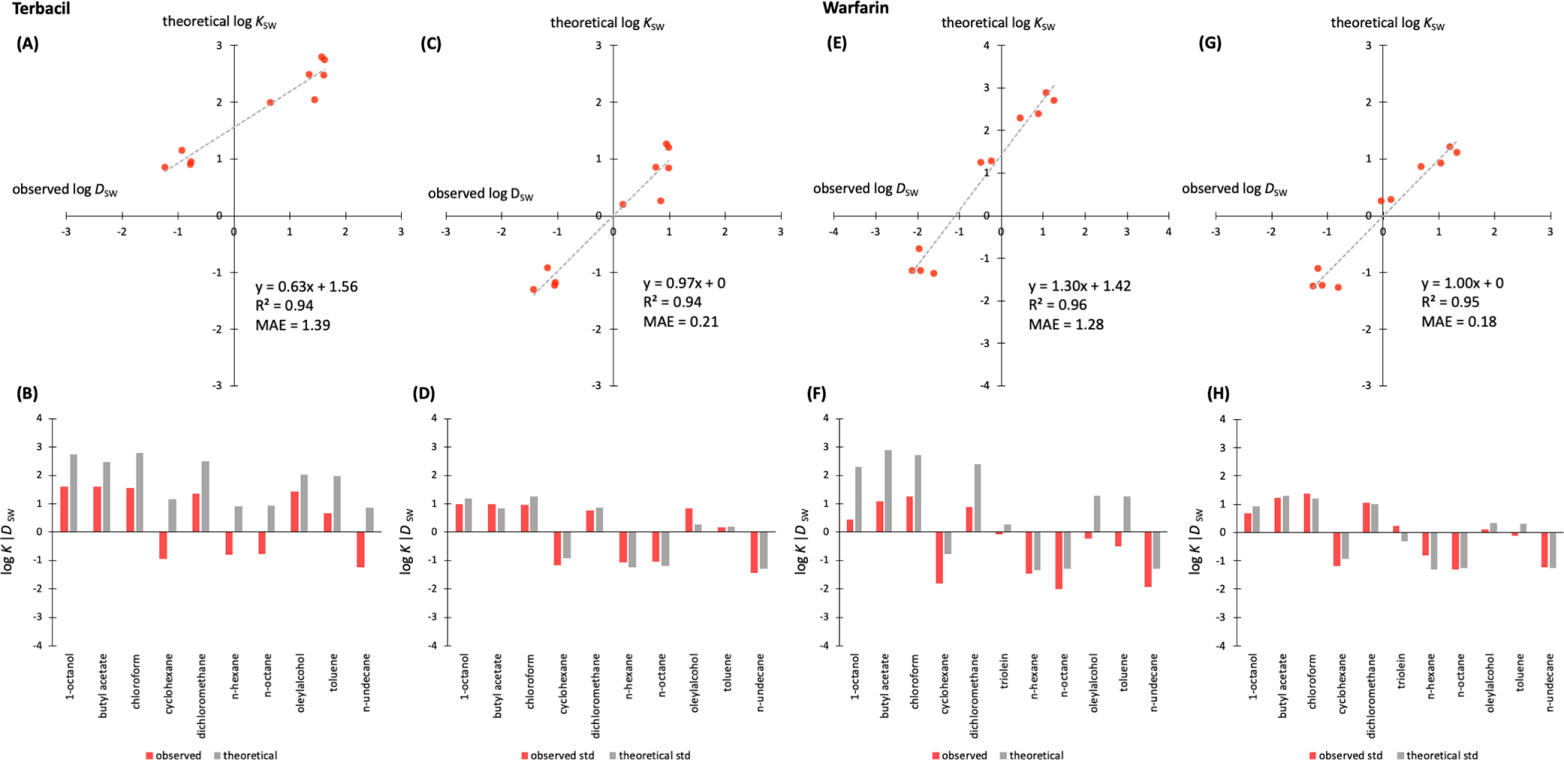
Observed log *D*_SW_ and theoretical log *K*_SW_ values for terbacil (A–D) and warfarin (E–H) for all 10 solvents before standardization (A and B for terbacil and E and F for warfarin) and after standardization (C and D for terbacil and G and H for warfarin) with a standard scaler. The standard scaler is described in the Materials and Methods section.

As described earlier in the methods, our theoretical understanding of the mechanisms controlling the partitioning of chemicals between organic solvents and water led us to the conclusion that while the absolute values of the distribution ratio of a chemical, e.g., *D*_octanol–water_ and *D*_hexane–water_ are going to be different at different pH values, e.g., pH = 2 and pH = 8, the differences between *D*_octanol–water_ and *D*_hexane–water_ are going to be the same (eq 6 – 10). This hypothesis was confirmed in our experimental observations (Figure 3). Figure 3 shows the observed log *D*_SW_ values for two examples, terbacil and warfarin, and their theoretical *K*_SW_ values before and after standardization with a standard scaler. We observed that standardizing did not affect the *R*^2^ between observed and theoretical values, but it did drastically reduce the MAE from 1.39 to 0.21 for terbacil and from 1.28 to 0.18 for warfarin. We also observed a change in the intercept of the trendline between observed and theoretical data for both examples (Figure 3), which dropped to 0 after standardization, and a change in the slope, which increased from 0.64 to 0.97 for terbacil and decreased from 1.30 to 1. In both cases, the intercepts were reduced to 0 and the slopes approximated 1. This observation could have important implications for future measurements of *D*_SW_ and *K*_SW_ for environmental chemicals when trying to evaluate their environmental behavior. For example, let us assume that one is trying to characterize the following physicochemical properties for a given chemical: the octanol–water equilibrium partition ratio (*K*_OW_), the organic carbon–water equilibrium partition ratio (*K*_OC_) and their corresponding distribution ratios at pH = 5 (*D*_OW_^pH=5^ and *D*_OC_^pH=5^). Since *D*_OC_^pH=5^ can be described as
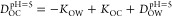
11one would only need to determine the *K*_OW_, *K*_OC_, and *D*_OW_^pH=5^ to calculate *D*_OC_^pH=5^. To the best of our knowledge, this is the first study to report this observation.

**Figure 4:**
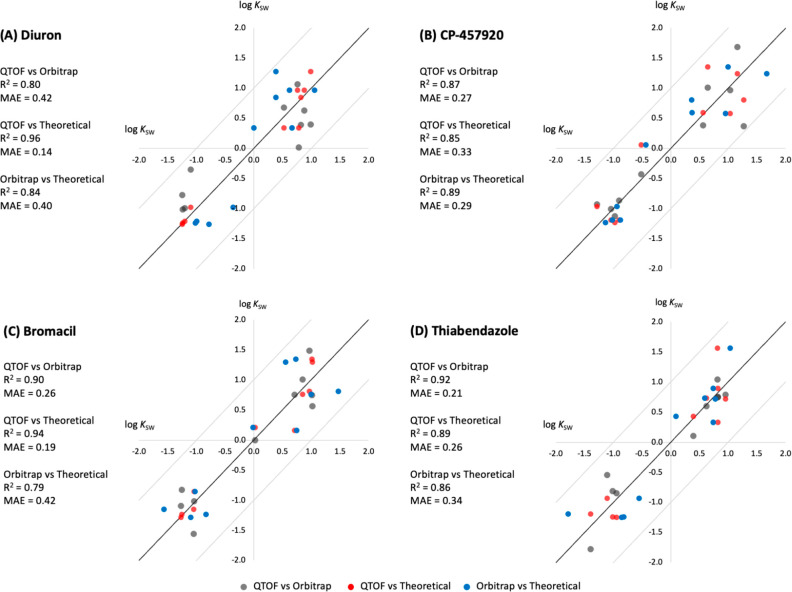
Four examples of the 16 chemicals whose log *K*_SW_ that showed an agreement of *R*^2^ > 0.8 between QTOF and Orbitrap methods. The black diagonal line is the 1-to-1 agreement line, and the two gray diagonal lines are the +1 and −1 log unit deviation lines. The figure shows log *K*_SW_ after standardizing with the standard scaler.

The log *K*_SW_ values (*D*_SW_ after standardization) that were observed with the QTOF method generally showed better agreement with the theoretical values from the UFZ-LSER database (average *R*^2^ = 0.92) than the log *K*_SW_ values from the Orbitrap method with the theoretical values from the UFZ-LSER database (average *R*^2^ = 0.85) (Table S1 and Figure 4). This observation was also reflected in the absolute errors (or absolute differences) between the QTOF log *K*_SW_ values and the theoretical values (average MAE = 0.22) and between the Orbitrap log *K*_SW_ values and the theoretical values (average MAE = 0.33) (Figures S1A and S1B). The absolute errors (or absolute differences) were shown to vary by partitioning system (Figures S1C and S1D). Butyl acetate and oleyl alcohol showed the largest errors (median values) in the QTOF method, while *n*-hexane and *n*-octane showed the largest errors (median values) in the Orbitrap method (Figures S1C and S1D).

### 3.3 RDKit Fragments

During the training and testing of the model, the cross-validation R^2^ for the predicted RDKit fragments in the training set ranged from 0.78 to 1 and the MAE ranged from 0.02 to 0.19 (Figure S2). The cross-validation *R*^2^ for the predicted RDKit fragments in the testing set ranged from 0.42 to 0.99 and the MAE ranged from 0.03 to 0.22 (Figures S3–S4).

When examining the predictions for the 16 chemicals in the mixture (Table S1), the larger absolute errors in the *K*_SW_ calculated with the Orbitrap method also culminated in larger errors in the predictions of RDKit fragments for the 16 chemicals (Figures S1E and S1F). The MAE for the RDKit fragment predictions with the QTOF method was 1.93, while with the Orbitrap method it was 3.62 (Figures S1E and S1F). Among the RDKit fragments for which we observed the largest errors in the QTOF method were aromatic N, amines, benzene rings, and bicyclic groups (Figure S1E). These RDKit fragments also showed large errors for the Orbitrap method (Figure S1F) in addition to CO bonds, CO bonds but not in the COO groups, and amides.

**Figure 5:**
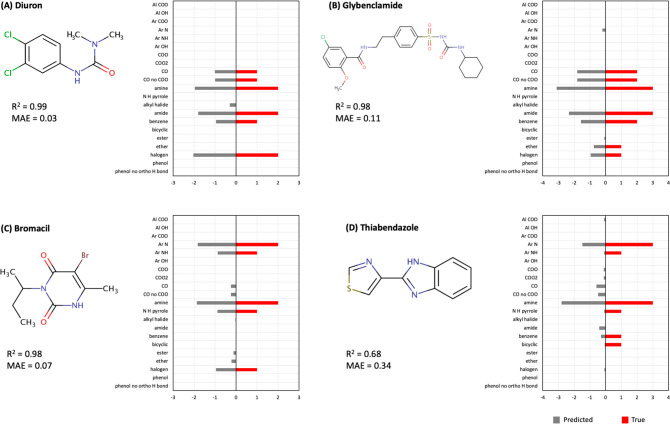
Examples of predicted and true RDKit fragments for four chemicals from the QTOF method. These four chemicals are a subgroup of the 16 chemicals whose log *K*_SW_ showed an agreement of *R*^2^ > 0.8 between the QTOF and Orbitrap methods. The predicted RDKit fragments are shown in gray and the true RDKit fragments are shown in red.

**Figure 6:**
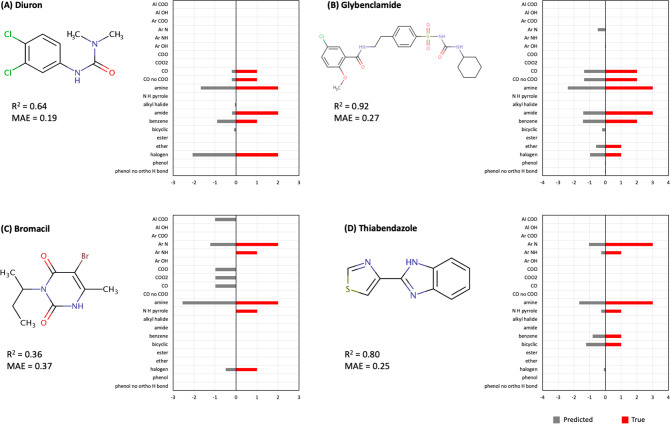
Examples of predicted and true RDKit fragments for four chemicals from the Orbitrap method. These four chemicals are a subgroup of the 16 chemicals whose log *K*_SW_ showed an agreement of *R*^2^ > 0.8 between the QTOF and Orbitrap methods. The predicted RDKit fragments are shown in gray, and the true RDKit fragments are shown in red.

When comparing predicted RDKit fragments to true RDKit fragments, it is important to note that while the true fragments are expressed as integers (e.g., 2 amines and 1 benzene ring), the predicted fragments are presented as decimals because they represent probabilities (e.g., 2.2 amines and 0.3 benzene rings; Figures 5 and 6). When matching to true fragments, it is thus recommended to use decimals to calculate *R*^2^ as a metric of similarity instead of rounding the predicted values to the nearest integer. Rounding can introduce errors in the predictions that ultimately can lead to unnecessarily erroneous matches.

The predicted RDKit fragments from the QTOF method showed overall better agreement with the true RDKit fragments compared with the predicted fragments from the Orbitrap method. The calculated *R*^2^ between true and predicted RDKit fragments showed an average of 0.71 for the QTOF method and an average of 0.47 for the Orbitrap method (Table S2). The MAE was on average 0.23 for the QTOF method and 0.31 for the Orbitrap method (Table S2).

When comparing the predictions for four example chemicals in Figures 5 and 6, we see that while both methods were in relative agreement about the presence of a given RDKit fragment, the QTOF method showed higher accuracy at also predicting the right number of that fragment. Four additional examples are presented in Figure S5 and S6. Similar observations made for the chemicals in Figures 5 and 6 can also be seen in Figure S5 and S6. The comparisons for all 16 chemicals are presented in Supplemental Spreadsheet 4.

For most chemicals, the predictions based on the Orbitrap method were less accurate than the predictions based on the QTOF method. As the differences between the QTOF and the Orbitrap methods were consistent, one has to wonder what the reason behind these discrepancies could be. Given that the software that was used to process the raw datafiles from the two instruments was the same (MS-Dial) with very similar parameters for the two data sets (Supplementary Spreadsheet 3), it is unlikely that the observed differences in *K*_SW_ and RDKit fragments emerge as a result of the software processing. Additionally, given that the machine learning algorithm is agnostic as to the instrument type used for the analysis and considering that the errors are already present in the measurements of *K*_SW_, it is unlikely that these differences come from this part of the workflow. The data processing scripts after alignment with MS-Dial were the same for the two instruments with the exception of the mass difference filter between the detected monoisotopic mass and the theoretical monoisotopic mass. The mass difference filter for QTOF was set at 10 ppm whereas that for Orbitrap was set at 5 ppm. This seemed to have little effect on the QTOF data, as for 41 out of the 44 detected chemicals, the mass differences were already below 5 ppm (Supplementary Spreadsheet 3). Considering all these above-mentioned factors do not appear to constitute significant sources of error, it seems that the observed discrepancies are more likely due to differences on the hardware side than on the software side.

On the hardware side, one potential source of error is in the chromatography; however, given the similarity in the chromatography columns used in the two methods and the similarity in the gradient solvents, it is unlikely that the observed differences originate on the chromatography side. Considering the higher mass resolution and mass accuracy of the Orbitrap, one would expect that the observed *K*_SW_ values and thus the predictions with the Orbitrap method would show higher accuracies compared to those of the QTOF method if the driving factor was mass resolution or mass accuracy. Based on data from the manufacturers of the two instruments, Thermo Scientific^12^ and Agilent,^13^ an Orbitrap mass spectrometer is expected to have a mass resolution of 200,000 at an *m*/*z* of 300, while a QTOF is expected to have a mass resolution of 40,000 for the same *m*/*z*. The analysis of the samples with the Orbitrap method revealed more chemicals in the mixture compared to the QTOF method (113 vs 44) which is in line with the higher mass resolution of the Orbitrap; however, both the measurements of *K*_SW_ and the predictions of RDKit fragments contained larger errors in the Orbitrap method compared to the QTOF method.

One of the most likely explanations that we arrived at was that the observed differences stem from the differences in the intrascan dynamic range of the two instruments. Orbitraps have been previously shown to have a narrower intrascan dynamic range compared to QTOFs^14^ likely due to the limited physical capacity of the C-trap and Orbitrap components of the instrument. A narrower intrascan dynamic range would essentially mean a narrower range where the response of the instrument is linear relative to the concentration of the compounds in the solution. As mentioned earlier in our methods, eq 2 assumes that the analytes are within the linear range of the calibration curve. A narrower linear range would lead to an increased likelihood of the analyte being in the sigmoid edges of the calibration curve (lower or higher end). This would, in turn, affect the numerator and/or the denominator of eq 2 and would lead to erroneous calculations of *K*_SW_ and thus erroneous predictions of RDKit fragments.

Finally, another plausible reason behind the observed differences could be the use of polarity switching. In the Orbitrap method, we used polarity switching for positive and negative ionization, whereas in the QTOF method, we had to run the samples separately for each ionization mode, since the instrument does not offer that functionality. Polarity switching increases the cycle times of the instrument resulting in fewer data points across chromatographic peaks.^26^ Less refined chromatographic peaks can result in erroneous peak areas, which would in turn affect the calculations of *K*_SW_ and as a consequence the predictions of RDKit fragments.

It should be noted at this point that the purpose of the study is not to declare one method better than the other but to understand the extent to which our approach is reproducible across different platforms and which parameters may influence its accuracy and reproducibility. Considering the observations for the two methods, at this stage we can only recommend the use of our method with a QTOF mass spectrometer.

### 3.5 Limitations and Future Considerations

One limitation that needs to be acknowledged is that our experiments use concentrations that are 10 or even 100 times higher than those found in biological or environmental samples. The application of our method in real-world samples would require a 10- or 100-fold concentration of the samples and potentially a cleanup step with a solid phase extraction (SPE) column to remove some of the matrix in order to reach levels that are within the detectable range and within the linear range of the calibration curve. Our follow-up study will focus on evaluating the application of the method in environmental and biological samples.

Another limitation is that our model is trained on equilibrium partition ratios that are calculated using poly parameter linear free-energy relationships (PP-LFERs).^27,28^ Uncertainties associated with these calculations can in some cases exceed 1 log unit; however, the overall average errors appear to be smaller. For instance, comparing experimentally determined partition ratios between octanol and water (*K*_OW_) to PP-LFER calculated values for a set of 75 chemicals, Tülp et al.^28^ observed a root-mean-squared error (RMSE) of 0.72 log units.

While these errors are critical in determining the equilibrium partition ratios of chemicals in order to understand their environmental fate and behavior, for the purposes of our study, these errors are of smaller importance. The purpose of our workflow is not to provide a set of accurate measurements of equilibrium partition measurements, but rather to utilize hybrid measurements-predictions in order to propose candidate structures for detected chemical features in NTA. Misassigned structures is a logistical possibility that needs to be considered; however, misassigned structures can also occur in MS/MS spectra matching. Since confirmation requires additional information from analytical standards or other *in silico* approaches, these misassignments are not deemed to be of critical importance. We need at this point to clarify that our workflow alone is not meant to replace analytical standards on its own but rather to provide a layer of evidence, which in combination with other layers of evidence from independent sources can help elucidate the molecular structures of detected chemical features in NTA. While analytical standards have long been the gold standard of identification, it is important to also acknowledge that there is a need to explore and deploy alternative approaches when analytical standards are not available. MS/MS fragmentation and matching with *in silico* generated spectra is another approach aiming to tackle the lack of analytical standards and can be combined with our approach in order to support chemical identification.

All limitations considered, our approach, nevertheless, showed great promise in characterizing molecular structures of chemical features detected through NTA. Our approach presents a new angle in structure elucidation for NTA that can be combined with other computational approaches such as MetFrag,^29−31^ CFM-ID,^32^ MS-DIAL,^17^ and MS-FINDER^17^ to assist in deriving molecular structures.

## Supporting Information Available

Tables S1–S2 and Figures S1–S6 (PDF)
Supplementary Spreadsheet 1: Chemical mixture—ENTACT 504—chemicals and structures (XLSX)
Supplementary Spreadsheet 2: TurboChemDB—The database with the theoretical *K*_SW_ values used to train the machine learning model (XLSX)
Supplementary Spreadsheet 3: *K*_SW_ measurements and underlying data from the QTOF and Orbitrap methods(XLSX)
Supplementary Spreadsheet 4: RDKit fragment predictions for all chemicals from the two methods (XLSX)

## Notes

All code used in this study is available on GitHub under https://github.com/dimitriabrahamsson/turbo-chem.
