# Supplementary material for: Extracting Structural Information from Physicochemical Property Measurements Using Machine Learning—A New Approach for Structure Elucidation in Non-targeted Analysis
Source: Environ Sci Technol. 2023 Sep 25;57(40):14827–38. doi: 10.1021/acs.est.3c03003 (PMC10569036; doi:10.1021/acs.est.3c03003)
Supplement: Supplementary file 1 — es3c03003_si_001.pdf [file es3c03003_si_001.pdf]

Supporting Information

Extracting structural information from physicochemical property measurements using machine learning - A new approach for structure elucidation in non-targeted analysis

Dimitri Abrahamsson[1,2 *+], Christopher L. Brueck[3,4 *], Carsten Prasse[3,5], Dimitra A. Lambropoulou[6,7], Lelouda-Athanasia Koronaiou[6,7], Miaomiao Wang[8], June-Soo Park[2,8] and Tracey J. Woodruff[2]

[1] Department of Pediatrics, New York University Grossman School of Medicine, New York, NY 10016, USA

[2] Department of Obstetrics, Gynecology and Reproductive Sciences, Program on Reproductive Health and the Environment, University of California, San Francisco, CA 94107, USA

[3] Department of Environmental Health and Engineering, Johns Hopkins University, Baltimore, MD 21205, USA

[4] Exponent, Environmental and Earth Sciences Practice, Bellevue, WA 98007, USA

[5] Risk Sciences and Public Policy Institute, Bloomberg School of Public Health, Johns Hopkins University, Baltimore, MD, 21205, USA

[6] Department of Chemistry, Aristotle University of Thessaloniki, University Campus, 54124 Thessaloniki, Greece

[7]Center for Interdisciplinary Research and Innovation (CIRI-AUTH), Balkan Center, Thessaloniki, 57001, Greece

[8] Department of Toxic Substances Control, Environmental Chemistry Laboratory, California Environmental Agency, Berkeley, CA 94710, USA

*These two authors contributed equally

[+]Corresponding author: Dimitri Abrahamsson, dimitri.abrahamsson@gmail.com

Supporting information: pages S1-S9, tables S1-S2 and figures S1-S6

Table S1: Compounds whose log K_{SW} showed good agreement between the QTOF and Orbitrap methods. The table shows the associations of the log K_{SW} between QTOF and Orbitrap, between QTOF and theoretical values, and between Orbitrap and theoretical values. The theoretical values are from the UFZ-LSER database.

	**QTOF vs Orbitrap**		**QTOF vs Theoretical**		**Orbitrap vs Theoretical**	
	R^2	MAE	R^2	MAE	R^2	MAE
Glybenclamide	0.93	0.23	0.97	0.14	0.90	0.26
2,4-Dihydroxybenzophenone	0.88	0.28	0.97	0.13	0.88	0.29
Diuron	0.80	0.42	0.96	0.14	0.84	0.40
Warfarin	0.81	0.38	0.95	0.18	0.76	0.39
Leflunomide	0.91	0.28	0.95	0.17	0.94	0.22
Bromacil	0.90	0.26	0.94	0.19	0.79	0.42
N-Phenyldiethanolamine	0.95	0.22	0.94	0.21	0.96	0.30
Terbacil	0.91	0.26	0.94	0.21	0.86	0.32
4-Chlorophenylurea	0.84	0.32	0.92	0.22	0.79	0.42
Azathioprine	0.93	0.24	0.91	0.23	0.94	0.23
Fuberidazole	0.92	0.25	0.90	0.26	0.76	0.43
Thiabendazole	0.92	0.21	0.89	0.26	0.86	0.34
Carbamazepine	0.85	0.34	0.87	0.24	0.82	0.32
CI-1044	0.95	0.18	0.85	0.32	0.91	0.26
CP-457920	0.85	0.27	0.85	0.33	0.89	0.29
Furalaxyl	0.83	0.37	0.84	0.32	0.75	0.38
Average	0.89	0.28	0.92	0.22	0.85	0.33

Table S2: R^2 and MAE for the predicted and true RDKit fragments from the QTOF and Orbitrap methods.

	QTOF R^2	QTOF MAE	Orbitrap R^2	Orbitrap MAE
Diuron	0.99	0.03	0.60	0.21
N-Phenyldiethanolamine	0.00	0.35	0.23	0.22
Azathioprine	0.92	0.48	0.99	0.25
Warfarin	0.30	0.31	0.46	0.24
Furalaxyl	0.74	0.16	0.00	0.60
4-Chlorophenylurea	0.79	0.16	0.04	0.39
Glybenclamide	0.98	0.11	0.92	0.24
CP-457920	0.64	0.37	0.75	0.30
Fuberidazole	0.81	0.15	0.41	0.27
Thiabendazole	0.68	0.34	0.70	0.26
CI-1044	0.92	0.15	0.88	0.23
2,4-Dihydroxybenzophenone	0.83	0.25	0.13	0.45
Leflunomide	0.93	0.14	0.83	0.22
Bromacil	0.98	0.07	0.20	0.42
Terbacil	0.18	0.34	0.03	0.37
Carbamazepine	0.69	0.26	0.43	0.36
Average	0.71	0.23	0.47	0.31

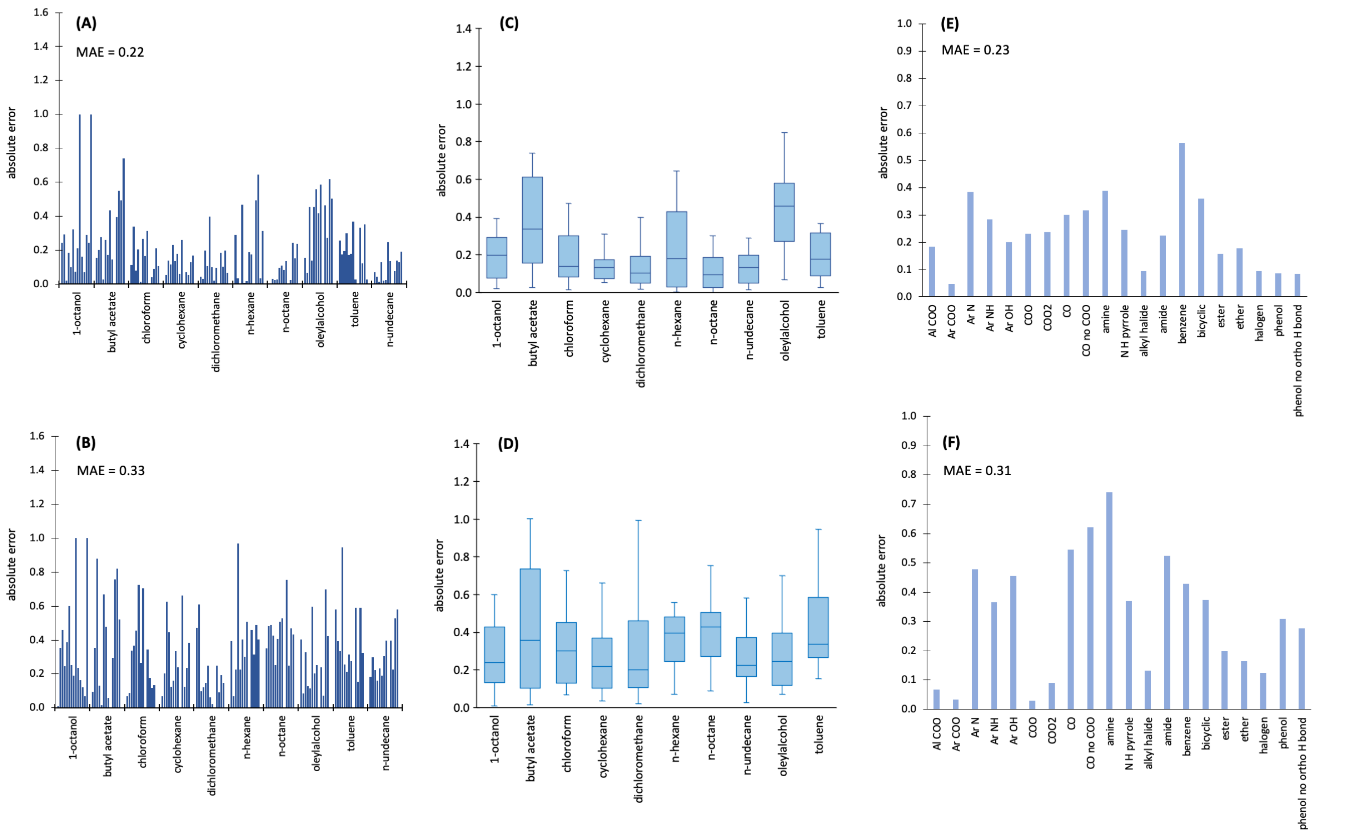

Figure S1: Absolute errors (or absolute differences) between observed and theoretical equilibrium partitioning ratios for the QTOF (A) and Orbitrap (B) methods as single chemicals and (C) and (D) grouped by solvent. The figure also shows the absolute errors (or absolute differences) between predicted and true RDKit fragments for the QTOF (E) and Orbitrap (F) methods. The comparisons here are focused on the 14 chemicals whose log K_{SW} that showed an agreement of $R^2 > 0.8$ between QTOF and Orbitrap methods.

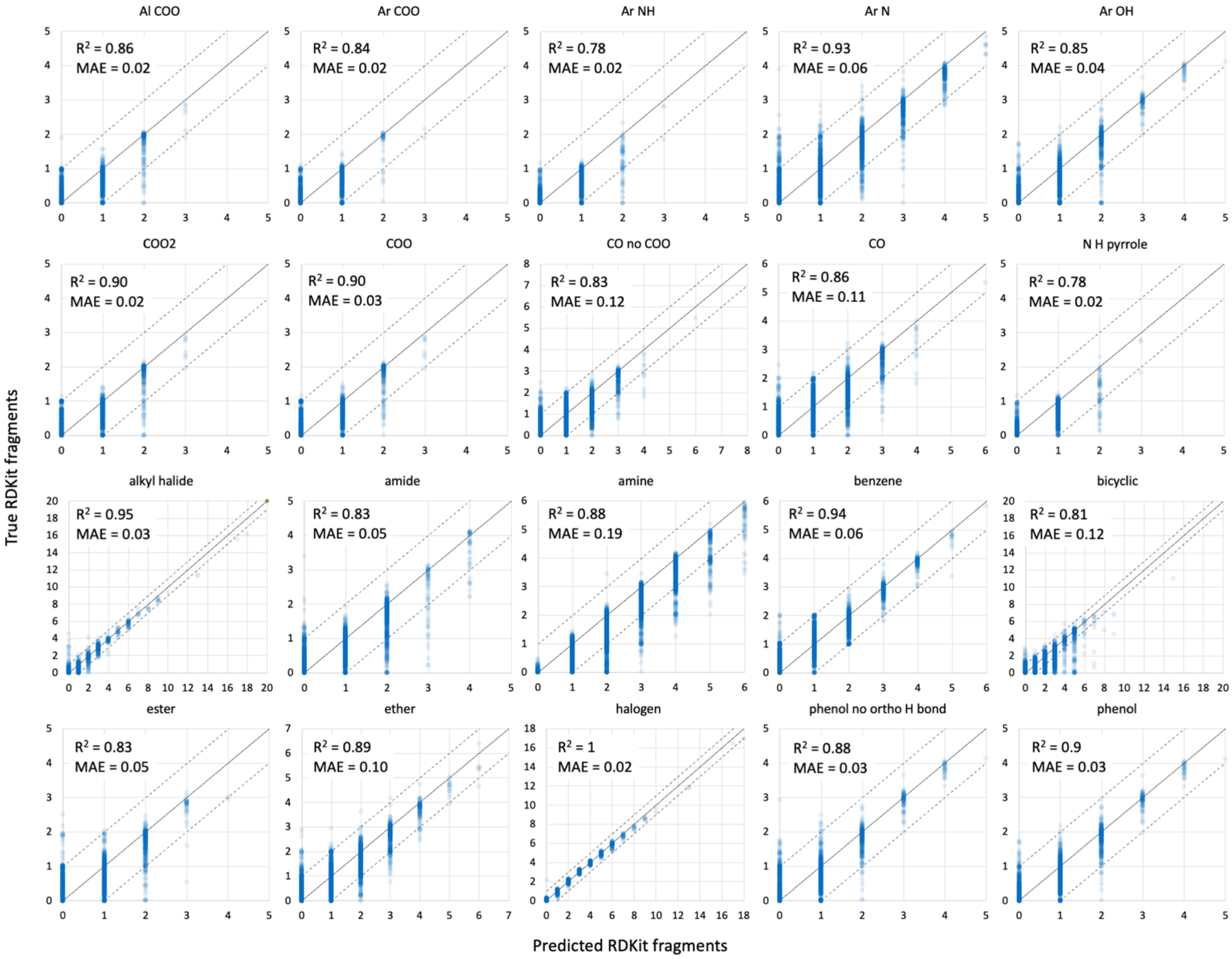

Figure S2: True and predicted RDKit framents for the training set after a 5-fold cross validation. R^2 is the cross-validation coefficient of determination and MAE is the mean absolute error of the five iterations.

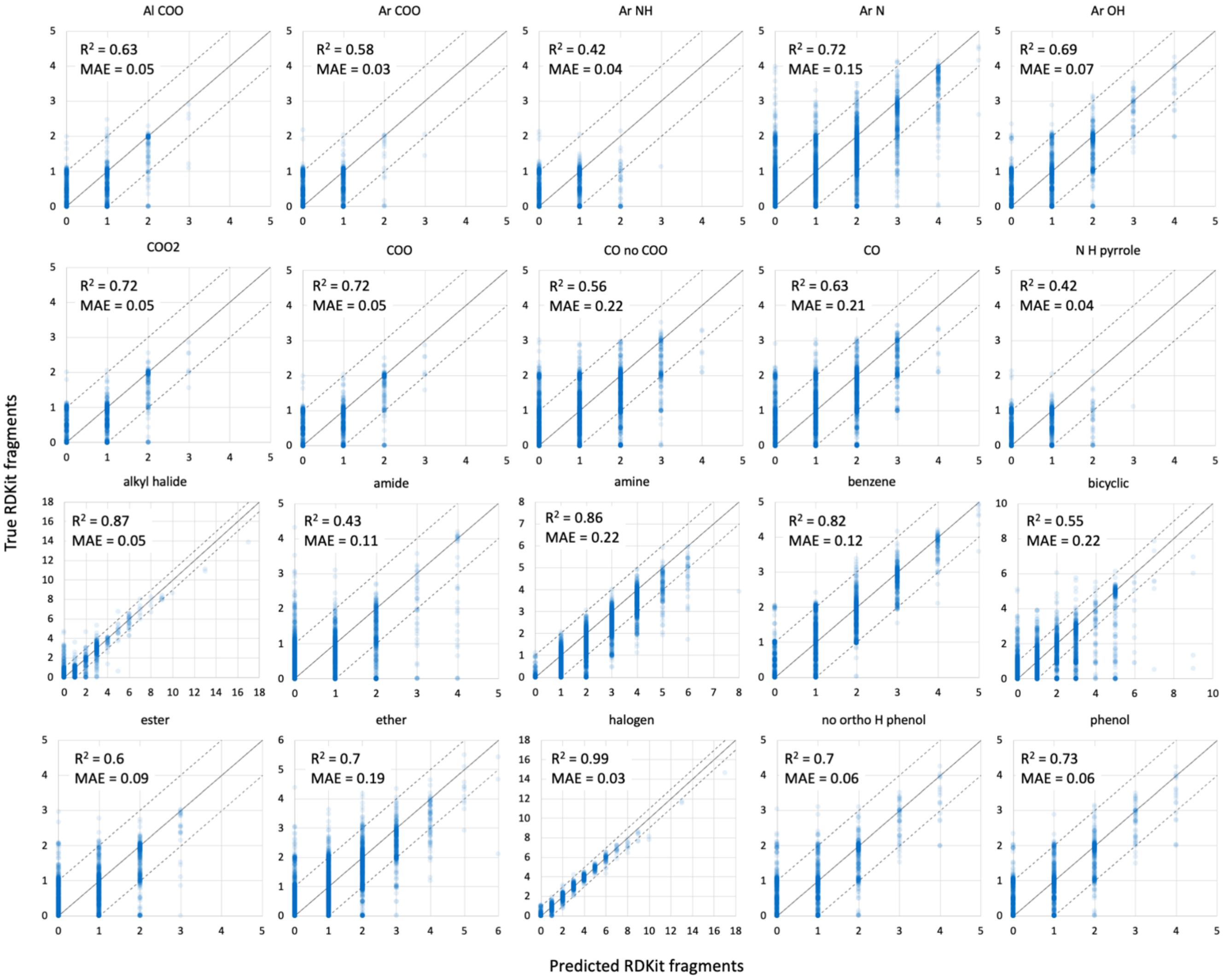

Figure S3: True and predicted RDKit framents for the testing set after a 5-fold cross validation. R^2 is the cross-validation coefficient of determination and MAE is the mean absolute error of the 5 iterations.

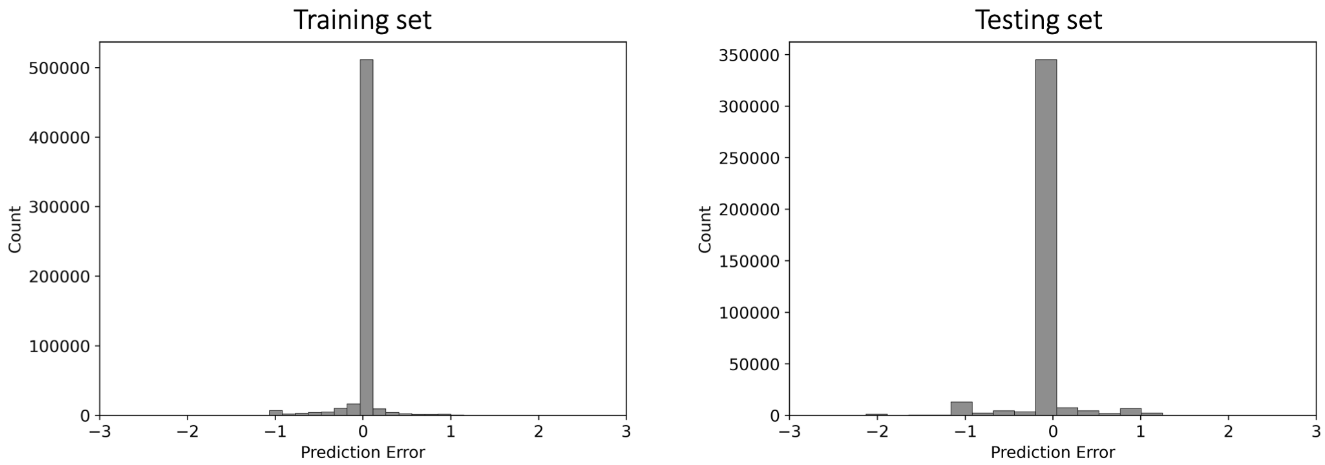

Figure S4: Errors in the predictions of the RDKit fragments for the chemicals in the training and testing sets during the 5-fold cross-validation.

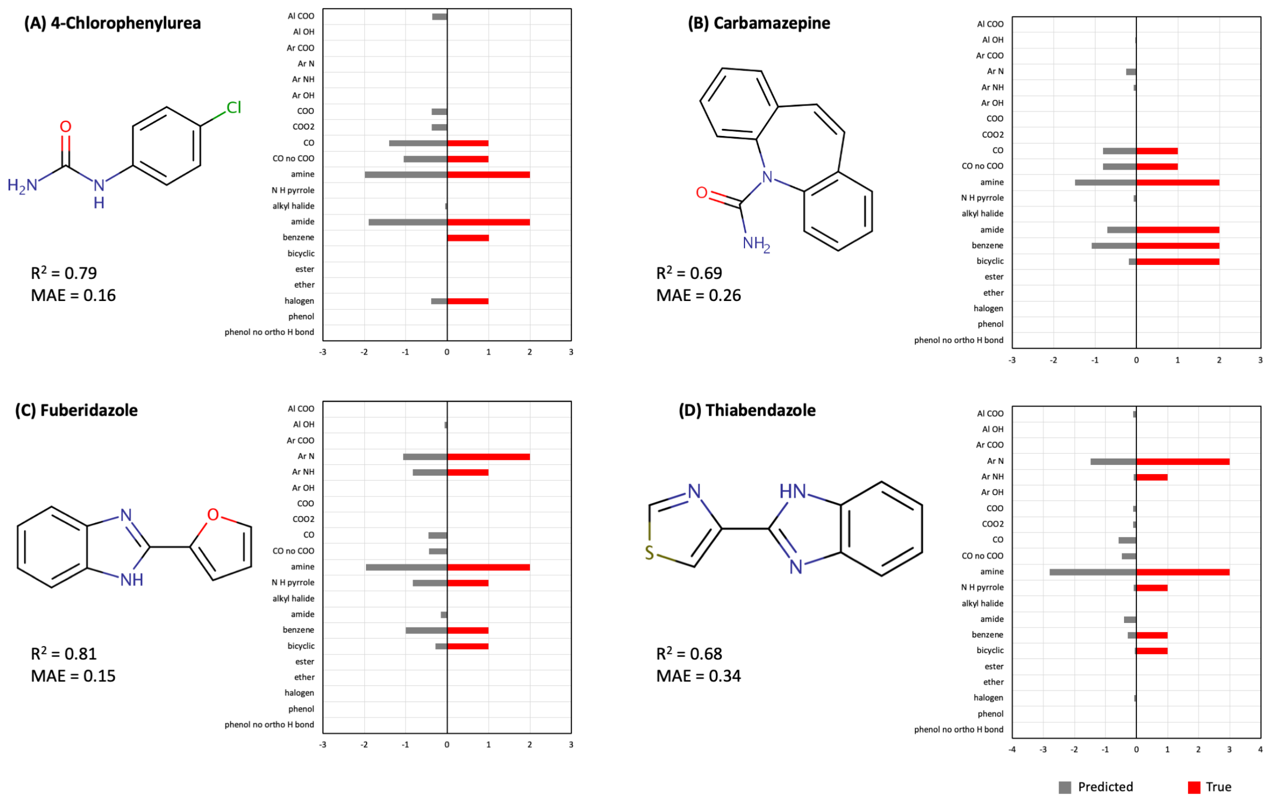

Figure S5: Examples of predicted and true RDKit fragments for four chemicals from the QTOF method. These four chemicals are a subgroup of the 14 chemicals whose log K_{SW} showed an agreement of of $R^2 > 0.8$ between the QTOF and Orbitrap methods. The predicted RDKit fragments are shown in grey and the true RDKit fragments are shown in red.

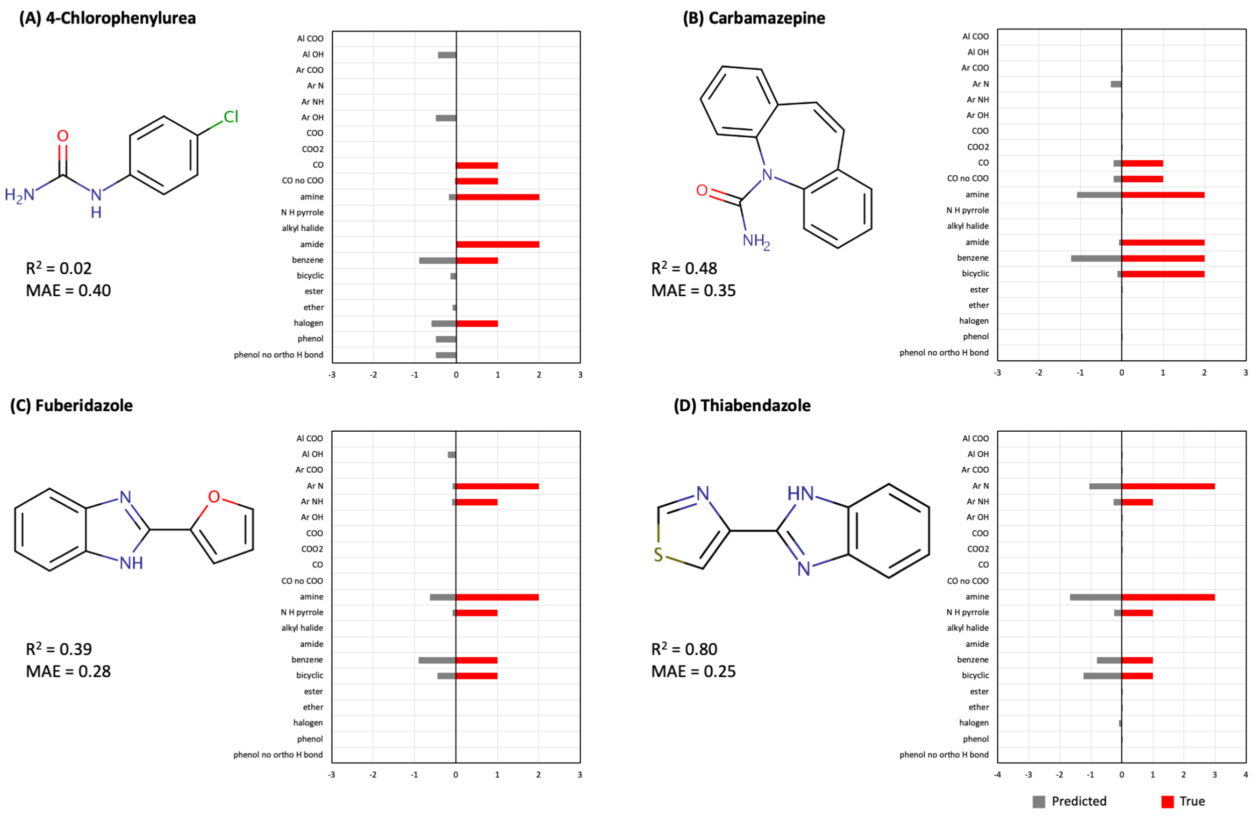

Figure S6: Examples of predicted and true RDKit fragments for four chemicals from the Orbitrap method. These four chemicals are a subgroup of the 14 chemicals whose log K_{SW} showed an agreement of of $R^2 > 0.8$ between the QTOF and Orbitrap methods. The predicted RDKit fragments are shown in grey and the true RDKit fragments are shown in red.
